# Kidney involvement in Bardet-Biedl syndrome: urinary concentrating defects highlight the major role of primary cilium in water reabsorption

**DOI:** 10.1186/2046-2530-1-S1-O5

**Published:** 2012-11-16

**Authors:** C de Melo, A Mockel, C Stoetzel, V Marion, H Dollfus

**Affiliations:** 1 Laboratoire Physiopathologie des Syndromes Rares Héréditaires, AVENIR-INSERM, France

## 

Ciliopathies are responsible for multiple organ dysfunctions with chronic kidney disease as one of cardinal clinical features. We studied the renal phenotype in 33 patients diagnosed with Bardet-Biedl Syndrome (BBS) and found renal abnormalities to be present in 82% of patients (27/33). An impaired urinary concentrating ability was the most frequent manifestation (63%, 19/30) in non dialyzed and non transplanted patients, which could be observed even in the absence of renal failure or cystic formation identified by ultrasonographic and magnetic resonance imaging. In order to specify the pathophysiology involved in this urinary concentrating defect, we focused on the role of the primary cilia and its interaction with the anti-diuretic hormone, arginine-vasopressin (AVP) as it was previously shown that the AVP-Receptor-2 (AVPR2) was localized on the primary cilia. To do so, we studied the effects of vertebrate-specific chaperonin-like proteins (BBS6, 10 and 12) inactivation in cultured cell line HCD (Human Collecting Duct) by way of RNA interference techniques. Our results show that chaperonin-like proteins deprivation in vitro leads to primary cilia loss in HCD cells, resulting in an inability to detect luminal AVP and to activate the targeting of aquaporin-2 (AQP-2) to the apical membrane of the renal epithelial cell, thus unable to absorb water. Interestingly, water reabsorption through restored targeting of AQP-2 was achieved by forskolin treatment – a receptor-independent adenylate cyclase activator – demonstrating that intracellular machinery was present but not activated. This study highlights the major role of primary cilia in efficient water reabsorption in the collecting duct.

